# Characterization analysis and heavy metal‐binding properties of *CsMTL3* in *Escherichia coli*

**DOI:** 10.1002/2211-5463.12520

**Published:** 2018-09-19

**Authors:** Xing Xu, Ling Duan, Jingwen Yu, Chenggang Su, Jinhua Li, Dan Chen, Xingguo Zhang, Hongyuan Song, Yu Pan

**Affiliations:** ^1^ Key Laboratory of Horticulture Science for Southern Mountainous Regions Ministry of Education Southwest University Chongqing China; ^2^ College of Horticulture and Landscape Architecture Southwest University Chongqing China; ^3^ Comprehensive Testing Center of Guangzhou Entry‐Exit Inspection & Quarantine Bureau Guangzhou China

**Keywords:** *CsMTL3*, *Escherichia coli*, heavy metal tolerance, heavy metal‐binding, metallothionein

## Abstract

Members of the metallothionein (MT) superfamily are involved in coordinating transition metal ions. In plants, MT family members are characterized by their arrangement of Cys residues. In this study, one member of the MT superfamily, *CsMTL3*, was characterized from a complementary DNA (cDNA) library from young cucumber fruit; CsMTL3 is predicted to encode a 64 amino acid protein with a predicted molecular mass of 6.751 kDa. Phylogenetic analysis identified it as a type 3 family member as the arrangement of N‐terminal Cys residues was different from that of MT‐like 2. Heterologous expression of *CsMTL3* in *Escherichia coli* improved their heavy metal tolerance, particularly to Cd^2+^ and Cu^2+^, and led to increased uptake of Cd^2+^ and Cu^2+^; increased uptake was also observed for cells expressing *Arabidopsis thaliana* metallothionein 3 (AtMT3) and phytochelatin‐like (PCL), with greatest uptake in PCL‐expressing cells. These findings demonstrate that *CsMTL3* can improve metal tolerance, especially for Cd^2+^ ions, when heterologously expressed in *E. coli*, and suggest that the composition and arrangement of N‐terminal Cys residues are associated with binding capacity and preference for different metal ions.

**Abbreviations**
AtMT3: *Arabidopsis thaliana* metallothionein 3
CsMTL3: *Cucumis sativus*, metallothionein‐like protein type 3
MT: metallothionein
PCL: phytochelatin‐like
PC: phytochelatin

Diverse heavy metals, including copper and zinc, are essential micronutrients in many plant physiological processes. However, high concentrations, as well as nonessential heavy metal ions such as cadmium and mercury, can be toxic to living cells. To counter this toxicity, plants have evolved a suite of mechanisms for the chelation and sequestration of heavy metals. Phytochelatins (PCs) and metallothioneins (MTs) are the most well‐characterized heavy metal‐binding ligands in plants. Numerous reports have shown that MTs play crucial roles in maintaining metal homeostasis and protect against heavy metal toxicity through intracellular sequestration 1, 2, 3, 4, 5.

The MTs are a group of low molecular weight (7–10 kDa) proteins first discovered in the late 1950s. MTs are highly enriched in cysteine (Cys) residues 6, 7 with metal‐binding motifs (Cys‐Cys, Cys‐X‐Cys, or Cys‐X‐X‐Cys) that provide sulfhydryl ligands for coordinating bivalent metal ions 8, 9. They have been grouped into different classes according to the arrangement of their Cys residues 6, 10, 11, 12, 13, 14, 15, 16. MTs that contain 20 highly conserved Cys residues were classified as class I MTs and are generally found in vertebrates 17, 18. Class II MTs have a flexible arrangement of cysteines and are widely distributed not only in animals, but also in plants, fungi, and cyanobacteria 6, 13, 16, 19. Class III MTs encompass the phytochelatins (PCs) which are characterized by the structure (γ‐Glu‐Cys)*n*‐Gly (*n* = 2–11) 20, 21. Numerous evidences showed that class II MTs have also been identified in plants, but they have more diverse amino acid sequences and Cys residue arrangements than the mammalian MTs 6, 22, 23. In angiosperms, MTs and MT‐like proteins are further divided into four types 6, 24, based on the tissues in which they are expressed. Type 1 MTs are mainly expressed in roots 4, 25, type 2 MTs in leaves 26, 27, 28, type 3 MTs in ripening fruits and leaves, and type 4 MTs in ripening fruits and developing seeds 24. MTs have been characterized in *Oryza sativa* (rice) 29, *Arabidopsis thaliana* 24 *, Citrullus lanatus* (watermelon) 30, *Solanum lycopersicum* (tomato) 31, *Brassica juncea* 32, and *Cucumis sativus* (cucumber) 33. The tolerance of plants to bivalent metal ions [zinc (Zn), copper (Cu), cadmium (Cd), and mercury (Hg)] can be enhanced by over‐ or heterologously expressing plant MT genes 13. Moreover, some plant MT genes have been heterologously expressed in *Escherichia coli*, where they also increased tolerance to metals 1, 14, 29, 34. MTs have additionally been shown to play important roles in regulating gene expression and cell metabolism as well as scavenging reactive oxygen species 2, 9, 24.

In this study, we describe the isolation and characterization of a type 3 MT gene from *C. sativus*, metallothionein‐like protein type 3 (CsMTL3), which had a novel arrangement and number of cysteine (Cys) residues. The metal‐binding characteristics of CsMTL3 were investigated by heterologous expression in *E. coli*, where its metal accumulation was compared to *A. thaliana* metallothionein 3 (AtMT3) and phytochelatin‐like (PCL) to evaluate its metal‐binding properties. Our results show that *CsMTL3* is a candidate gene for improving metal tolerance in plants, especially for cadmium, and provides important insights for future studies of the function of *CsMTL3* in plants.

## Results

### Isolation and characterization of *CsMTL3*

**Figure 1:**
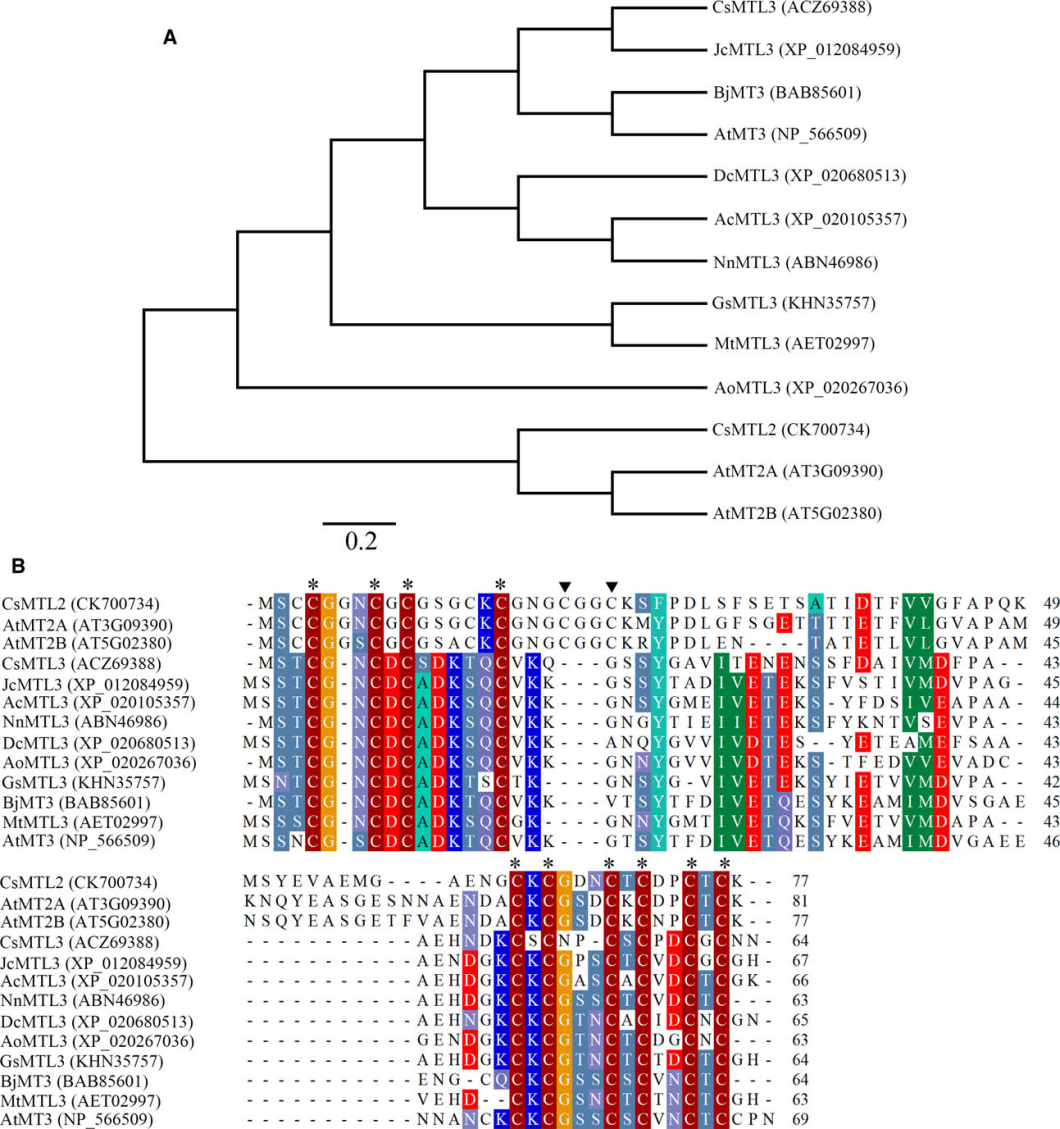
Phylogenetic tree of 10 selected plant MT proteins and amino acid alignment. (A) Phylogenic comparison of the amino acid sequences of plant MT proteins. Alignments were made in Clustal X using default parameters. Scale bar denotes 0.2 amino acids. Accession numbers for the MT proteins are included with the protein name. The MT genes are from *Arabidopsis* (At), *Jatropha curcas* (Jc), *Ananas comosus* (Ac), *Nelumbo nucifera* (Nn), *Dendrobium catenatum* (Dc), *Asparagus officinalis* (Ao), *Glycine soja* (Gs), *Brassica juncea* (Bj), *Medicago truncatula* (Mt), and cucumber (Cs). (B) Comparison of the amino acid sequence of CsMTL3 with other plant MT proteins. Amino acid residues that are conserved in at least eight of the eleven sequences are shaded, whereas those that are identical in all eleven proteins are in black. The stars indicate conserved cysteine residues, and arrowheads indicate the cysteines in C‐X‐X‐C motifs.

In this study, *CsMTL3* was identified from a cDNA library prepared from RNA from young cucumber fruits. It has an open reading frame of 234 bp and encodes a 64 amino acid polypeptide with a predicted molecular mass of 6.751 kDa. A phylogenetic tree for plant MTs was constructed based on amino acid sequences of MTs from *Jatropha curcas* (Jc), *Ananas comosus* (Ac), *Nelumbo nucifera* (Nn), *Dendrobium catenatum* (Dc), *Asparagus officinalis* (Ao), *Glycine soja* (Gs), *B. juncea* (Bj), *Medicago truncatula* (Mt), and *A. thaliana* (At). Analysis of this phylogenetic tree showed that CsMTL3 was different from CsMTL2 and could be classified as a type 3 MT (Fig. 1A). Multiple sequence alignment of these MT protein sequences showed that CsMT3 contains 10 cysteine residues, occurring as single C and C‐X‐C motifs in the N terminus and C‐X‐C in the C‐terminus, and showed high homology to type 3 MTs from other plant species (Fig. 1A). Further distinguishing the type 3 MTs from the type 2 MTs are a reduced number of cysteines and lack of C‐X‐X‐C motifs (Fig. 1B, arrowheads) in the type 3 MTs. The N‐ and C‐terminal cysteine‐rich regions are separated by a 39‐amino acid spacer devoid of cysteines (Fig. 1B).

### Expression of *CsMTL3* in response to metal stress

**Figure 2:**
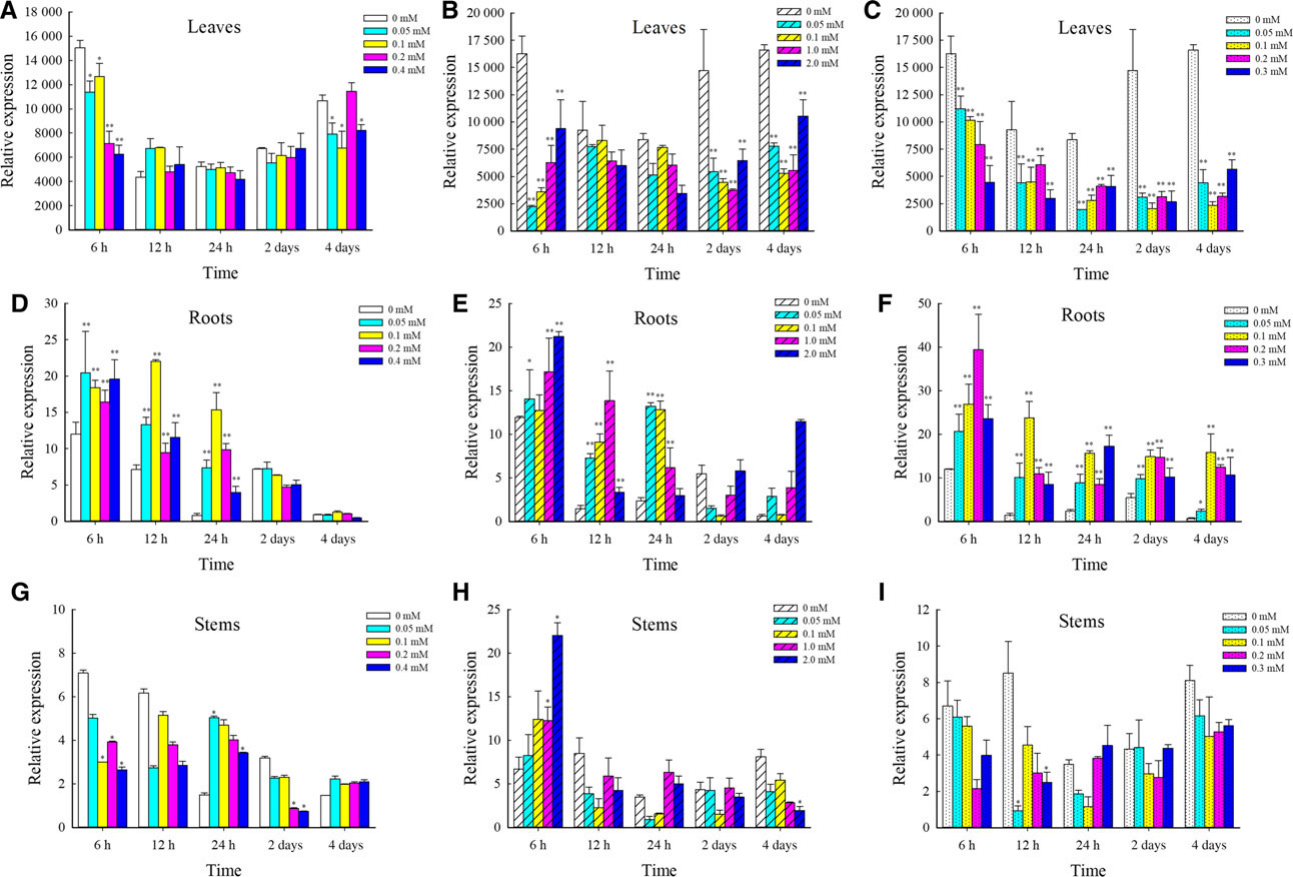
Relative expression levels of *CsMTL3* in the leaves, roots, and stems of cucumber plants grown under Cu^2+^, Zn^2+^, and Cd^2+^ stress. (A, D, and G) the expression of CsMTL3 in leaves, roots, and stems under Cu^2+^ stress; (B, E, and H) the expression of CsMTL3 in leaves, roots, and stems under Zn^2+^ stress; (C, F, and I) the expression of CsMTL3 in leaves, roots, and stems under Cd^2+^ stress. Values represent the mean ± SE of three biological replicates, with three technical replicates for each organ. Statistical significance was calculated with Student's *t*‐test: *P <* *0.05; and **P <* *0.01.

We used qPCR to evaluate the expression levels of *CsMTL3* from leaves, roots, and stems of plants exposed to increasing levels of Cu, Zn, and Cd ions. *CsMTL3* was most highly expressed in leaves (Fig. 2). While expression in leaves was reduced in response to all metal ions, Cd^2+^ stress showed the strongest reduction (Fig. 2A–C). Expression of *CsMTL3* in roots generally increased over time in response to metal stress (Fig. 2D–F), indicating that *CsMTL3* might enhance the tolerance of roots to heavy metal ions. Expression of *CsMTL3* in the stems was changed under metal ions stress, but there are no obvious differences among all the different metal concentrations (Fig. 2G–I). These results demonstrate that *CsMTL3* was most strongly expressed in leaves and that metal stress led to a decrease in expression in leaves and an increase in roots (Fig. 2).

### Expression and purification of *CsMTL3*,* AtMT3*, and *PCL* in *E. coli*

**Figure 3:**
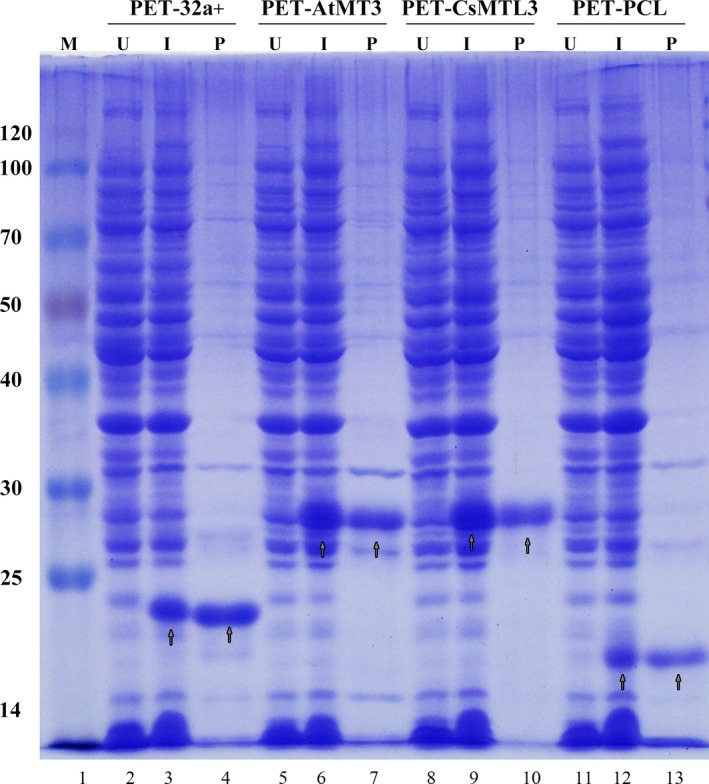
SDS/PAGE analysis of His‐tagged CsMTL3, AtMT3, and PCL fusion proteins heterologously expressed in *Escherichia coli*. Lane 1, molecular weight markers (kDa); lanes 2–13, proteins isolated from the transformed cells (pET32a (+), pET32a‐AtMT3, pET32a‐CsMTL3, and pET32a‐PCL). U, before IPTG induction; I, IPTG induction; and P, purified protein. The arrowheads represent the target proteins.

The predicted molecular weights for CsMTL3 and AtMT3 were 6.751 and 7.171, respectively. The proteins translated from His‐tagged CsMTL3 and AtMT3, and PCL as well as the His‐tag control were expressed in *E. coli* BL21 cells after induction with 1 mm IPTG (Fig. 3). In addition, the molecular weights of PCL and the His‐tag control were also consistent with the published report 33. The rights of the bands corresponding to per recombinant plasmid were indicated by the arrows (Fig. 3). The results showed that expression from the His‐tag control strain and PCL produced Trx‐His and Trx‐PCL proteins are 19.888 and 20.397 kDa, respectively (Fig. 3, lanes 3 and 12), which was absent in non‐induced cells (Fig. 3, lane 2). After induction of the Trx‐tagged CsMTL3 and AtMT3 strains, proteins of approximately 27.148 and 27.568 kDa in size, respectively, were produced (Fig. 3, lanes 6, 9, and 12), but Trx‐tagged CsMTL3, AtMT3, and PCL proteins were not displayed in the absence of IPTG (Fig. 3, lanes 5, 8, and 11).

### Metal tolerance in *E. coli* expressing fusion proteins

**Figure 4:**
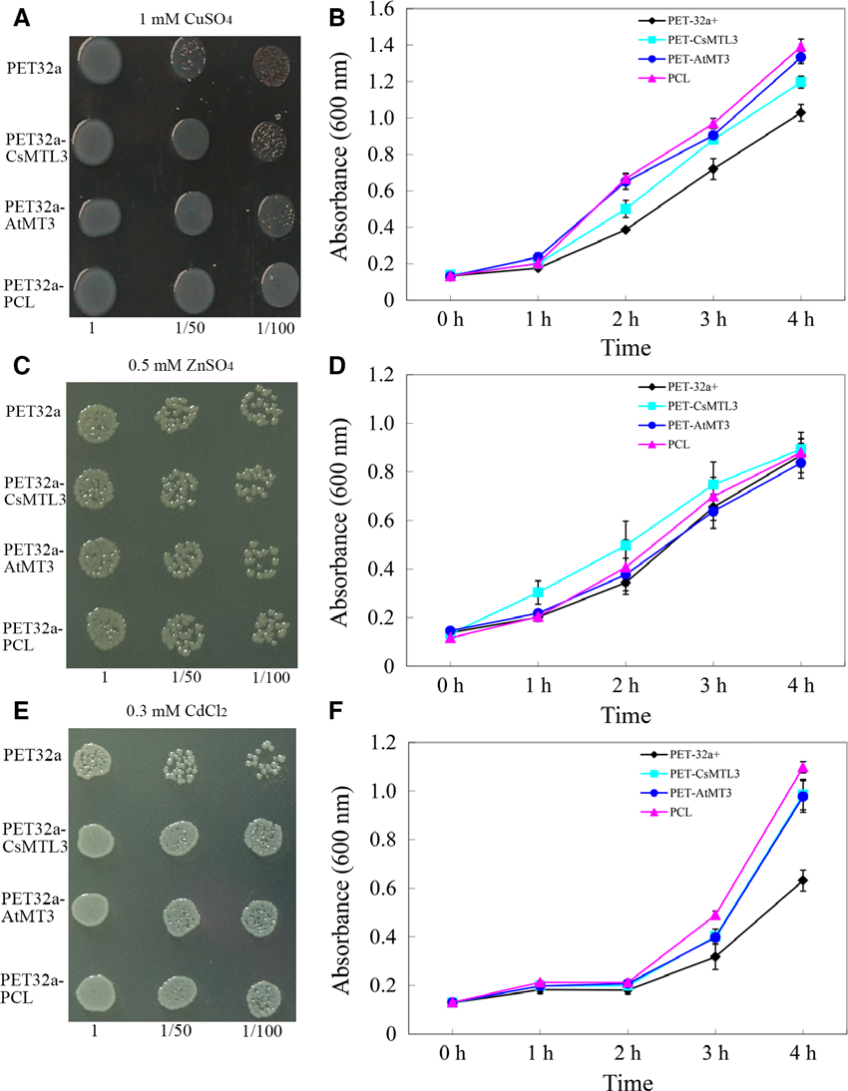
Growth of cells containing the recombinant plasmids (pET32a‐CsMTL3, pET32a‐AtMT3, and pET32a‐PCL) in the presence of different metal ions. Cells were grown as serial dilutions (1 : 1, 1 : 50, and 1 : 100) on LB agar under 1 mm CuSO_4_ stress (A), 0.5 mm ZnSO_4_ stress (C), and 0.3 mm CdCl_2_ (E) at 37 °C for 8 h. The growth curve of *Escherichia coli* cells was in liquid media supplemented with the 1 mm CuSO_4_ stress (B), 0.5 mm ZnSO_4_ stress (D), and 0.3 mm CdCl_2_ (F) at 37 °C for 4 h. The data are shown as mean ± standard error (SE) (*n* = 3). All assays were performed in triplicate.

To further explore the properties of CsMTL3, AtMT3, and PCL, *E. coli* BL21 cells containing the recombinant protein vectors were subjected to metal ion stress and their growth rate was measured (Fig. 4). No differences between the recombinant plasmids (pET32a‐CsMTL3, pET32a‐AtMT3, and pET32a‐PCL) and the control (pET32a (+)) were found when the cells were treated with ZnSO_4_ (Fig. 4B,C). By contrast, *E. coli* cells expressing the three MTs grew faster than controls in media containing CuSO_4_ (Fig. 4A,B) and CdCl_2_ (Fig. 4E,F) indicating that *CsMTL3*,* AtMT3*, and *PCL* increased tolerance to Cu^2+^ and Cd^2+^ ions, with the most marked increase for Cd^2+^ ions (Fig. 4C,D).

### Ion accumulation in *E. coli* expressing MT fusion proteins

**Figure 5:**
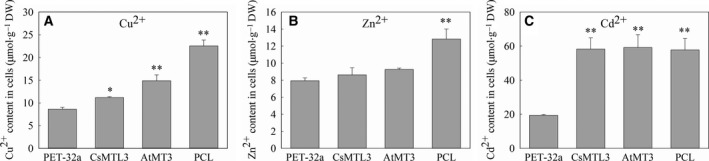
Metal ion accumulation in *Escherichia coli* heterologously expressing *CsMTL3*,* AtMT3*, and *PCL*. The copper (Cu), zinc (Zn), and cadmium (Cd) ion contents in *E. coli* were treated with 1 mm CuSO_4_ stress (A), 0.5 mm ZnSO_4_ stress (B), and 0.3 mm CdCl_2_ (C) for 3 h. Error bars represent standard error from at least 3 independent repetitions. Statistical significance was calculated with Student's *t*‐test: *P <* *0.05; and **P <* *0.01.

Previous results have shown that MTs bind metal ions, especially Zn^2+^ and Cd^2+^ 5, 32, 33, 34. Therefore, the three recombinant plasmids (pET32a‐CsMTL3, pET32a‐AtMT3, and pET32a‐PCL) were cultured in LB medium with IPTG to induce protein expression, to which Cu^2+^, Zn^2+^, or Cd^2+^ ions were added. Cells were harvested, and the concentration of accumulated metal ions (g^−1^ dry weight) was determined by flame atomic absorption spectrometry. While we found no significant difference in tolerance to Zn^2+^ (Fig. 4C,D), PCL showed a strong capacity for binding Zn^2+^ ions (Fig. 5B), consistent with published research 33. In contrast, all three of the MTs showed an increased accumulation of Cu^2+^ and Cd^2+^ compared to the control (Fig. 5A,C, *P* < 0.05 and *P *<* *0.01). This was especially true for Cd^2+^, where cells expressing CsMTL3 accumulated three times as much as the control (Fig. 5C). This specificity is different from CsMTL2, which accumulates more Zn^2+^ ions 33.

## Discussion

The Cys‐rich metal‐chelating MTs play an important role in metal homeostasis in many organisms 11, 35, 36, 37, 38, 39. Four types of MTs have been characterized in plants 6, 14. In this study, the MT‐like gene *CsMTL3* was isolated from young cucumber fruit. It is predicted to encode four Cys residues in the N terminus and six in the C‐terminus (Fig. 1B). Phylogenetic analysis indicates that CsMTL3 belongs to the type 3 MT family of plant proteins (Fig. 1A). In addition, the highly conserved CxCxxxCxCxxCxC motif of plant MTs was also found in CsMTL3 9. The type 2 MT family is characterized by conserved N‐terminal sequences, for example, MSCCGGN, MSCCGGS, MSSCCGGN, and MSCCSGN 9, 32, 33, 40, 41. By contrast, MSTCGN, MSSTCG, and MSSSCG sequences are found in the N terminus of the type 3 MTs (Fig. 1B), suggesting that this leading sequence might be involved in protein folding or stabilizing metal clusters 32, 33, 41.

The different expression patterns found in MTs may be linked to their biochemical and physiological functions 42, 43. For example, Cd^2+^ exposure led to a decrease in *CsMTL2* expression in leaves, roots, and stems, while Zn^2+^ had no effect 33. In another example, Cd treatment in *Phytolacca americana* inhibited *PaMT3‐1*, but induced *PaMT3‐2* and *PaMT3‐3* 43. In this study, *CsMTL3* expression was analyzed in different tissues from plants exposed to various concentrations of metal ions. It was most strongly expressed in leaves compared to stems and roots, consistent with the fact that type 3 plant MTs are more highly expressed in leaf mesophyll cells 24. However, *CsMTL3* expression was suppressed in leaves upon metal exposure, but induced in roots (Fig. 2). This increased expression in the roots could enhance the tolerance of cucumber plants to metal ions.

Plant MTs are also heavily involved in both metal metabolism and detoxification 20, 44. Due to the difficulty in isolating native MT proteins from plants, their metal‐binding properties are generally characterized by heterologous expression in bacteria 14, 33, 45, 46, 47. In this study, heterologous expression of CsMTL3 led to increased metal tolerance and metal ion accumulation, especially for Cu and Cd (Figs 4 and 5). These binding preferences are different from CsMTL2 33, consistent with the fact that the position and arrangement of Cys residues are crucial for the metal‐binding of MT proteins 45, 47, 48, 49. Plant MTs generally have two Cys‐rich regions, one each at the N‐ and C‐terminus 9. While we did not find obvious differences in the C termini of type 2 and 3 MTs, we found that the position and arrangement of N‐terminal Cys residues do differ (Fig. 1B). Furthermore, *CsMTL3* expression increased tolerance and accumulation of Cu and Cd ions (Figs 4 and 5), while *CsMTL2* showed a preference for Zn and Cd ions 33. We were also able to demonstrate significant differences in metal preference for different type 3 MTs (Figs 4 and 5). Taken together, these findings suggest that the composition and arrangement of N‐terminal Cys residues are associated with the preference and capacity of metal‐binding. Further studies will be required to decipher the underlying molecular mechanisms between *CsMTL2* and *CsMTL3* that lead to differences in metal accumulation.

## Materials and methods

### *CsMTL3* cloning and expression vector construction

The cDNA of *CsMTL3* (GenBank accession number: GQ487332) was characterized from a young cucumber fruit (*C. sativus* L. Cs0301) cDNA library according to the manufacturer's instructions (Clontech Laboratories, Inc., Mountain View, CA, USA). Using the homologous cloning method, the coding region of *CsMTL3* was amplified by polymerase chain reaction (PCR) with PrimeSTAR HS DNA polymerase (TaKaRa Biotechnology, Dalian, China) and the *CsMTL3* primer pair: forward, 5ʹ‐ATGTCGACATGTGGCAACTGCG‐3ʹ; reverse, 5ʹ‐CCGCTCGAGTCAATTGTTGCAGCCACAGTC‐3ʹ. After digested with *Xho*I, the *CsMTL3* cDNA coding region (195 bp) was subcloned into a pET32a (+) vector linearized with *EcoR*V and *Xho*I. The recombinant plasmid, pET32a‐CsMTL3, was confirmed by PCR, enzyme digestion, and DNA sequencing (Sangon Co., Shanghai, China). The *A. thaliana* metallothionein 3 gene (*AtMT3*, GenBank accession number: NM_112401.2) was PCR‐amplified from *A. thaliana* leaves using synthetic anchored primers: forward, 5ʹ‐ATGTCAAGCAACTGCGGAAGCTG‐3ʹ; reverse, 5ʹ‐CCGCTCGAGTTAGTTGGGGCAGCAAGTGCAGT‐3ʹ. The recombinant plasmid, pET32a‐AtMT3, was constructed and confirmed as described above. Construction of pET32a‐PCL was previously described 5, 33. In brief, Phytochelatin‐like (PCL) was designed and synthesized in plants according to the structure of phytochelatin (γ‐G1u‐Cys)11‐Gly 33, which had one of the bestl‐characterized heavy metal‐binding ligands in plant cells 6.

### Expression profiling of *CsMTL3* under Cd^2+^, Cu^2+^, and Zn^2+^ stress

Cucumber plants were cultured as previously described 33, and 6‐week‐old cucumber plants were grown for 0 h, 6 h, 12 h, 24 h, 2 d, and 4 d in the tray presence of CdCl_2_ (0.05, 0.1, 0.2, 0.3 mm), CuSO_4_ (0.05, 0.1, 0.2, 0.4 mm), and ZnSO_4_ (0.05, 0.1, 0.5, 1.0, 2.0 mm). Subsequently, all plant tissues were collected in the liquid nitrogen and stored at −70 °C until use. Total RNA was isolated using RNA prep Pure Plant Kit (TIANGEN, Beijing, China), and cDNA was synthesized with PrimeScript RT Reagent Kit with a gDNA Eraser (TaKaRa Biotechnology) according to the manufacturer's instructions. RT‐qPCR analysis was performed on a Bio‐Rad CFX96 Real Time System (Bio‐Rad Laboratories, Hercules, CA, USA) to detect the expression of *CsMTL3* using primers: forward, 5ʹ‐CAGCGGCCGAGCACAACGACAAGT‐3ʹ; reverse, 5ʹ‐GTGGGTGAAGAACAGAAATAAACA‐3ʹ. *CsActin* (GenBank accession number, DQ115883) was used as an internal control to evaluate relative gene expression levels with primers: forward, 5ʹ‐GGTGGTGAACATGTAACCTC‐3ʹ; reverse, 5ʹ‐TTCTGGTGATGGTGTGAGTC‐3ʹ. All samples were amplified in triplicate (three biological repeats with three technical repeats), and expression levels were calculated with the $2^{-\Delta \Delta C_{t}}$ method. Values are presented as the average ± standard error (SE) from three independent biological replicates.

### Expression and purification of recombinant *CsMTL3*,* AtMT3*, and *PCL*

The recombinant plasmids, pET32a‐CsMTL3, pET32a‐AtMT3, pET32a‐PCL, and empty vector pET32a (+), were transformed into *E. coli* strain BL21 (DE3) for expression of the recombinant proteins with Trx‐tagged. Protein expression and related assays were performed as previously described 33. These recombinant plasmid cells were grown at 37 °C to an OD_600_ of 0.6, and protein expression was induced with 1 mm isopropyl β‐d‐thiogalactoside (IPTG). After growing for an additional 3 h at 37 °C, cells were harvested by centrifugation. Recombinant proteins were purified with a MagneHis™ Protein Purification System (Promega, Shanghai, China) and analyzed by 15% sodium dodecyl sulfate/polyacrylamide gel electrophoresis (SDS/PAGE).

### Metal tolerance assay

*Escherichia coli* containing recombinant plasmids (pET32a‐CsMTL3, pET32a‐AtMT3, and pET32a‐PCL) or the control (pET32a (+)) were grown in liquid medium. Cultures were adjusted to OD_600_ = 0.5 and serial dilutions (1 : 1, 1 : 50, and 1 : 100), were spotted onto LB plates supplemented with 1 mm CuSO_4_, 0.5 mm ZnSO_4_, or 0.3 mm CdCl_2_, and then incubated for 8 h at 37 °C.

### Heavy metal‐binding assay

*Escherichia coli* containing recombinant plasmids (pET32a‐CsMTL3, pET32a‐AtMT3, and pET32a‐PCL) or the control (pET32a (+)) were induced with isopropyl β‐d‐1‐thiogalactopyranoside (IPTG) in 100‐mL flasks as described above for 1 h. Metal ions (CuSO_4_, ZnCl_2,_ and CdCl_2_) were then added to a final concentration of 300 μm. After 3 h, 50 mg cells were collected, placed in a 50 mL porcelain crucible, and heated in a muffle furnace to 500 ± 25 °C for 6 h. After cooling to room temperature, 1 mL nitric acid was added to the crucible. After the nitric acid evaporated, the crucible was again heated for 2 h in the muffle furnace. Finally, the remaining residues were dissolved with 10 mL 8.3% hydrochloric acid, and 8.3% hydrochloric acid was used as a negative control. This step was done in triplicate (three biological repeats with three technical repeats). The amount of metal bound by cells expressing the fusion proteins was analyzed by flame atomic absorption spectrometry as described 33.

### Statistical analyses

The metal‐binding assay was performed in triplicate, and standard error of the means was calculated. The data were statistically analyzed with Student's *t*‐test from the statistical analysis software spss 15.0 (IBM Corp, Armonk, NJ, USA). Differences were deemed significant at *P *<* *0.05.

## Author contributions

XX and YP conceived the study and drafted the manuscript. LD and JY performed the data mining and bioinformatics analysis. JY and JL carried out gene expression analysis. CS and XZ carried out reagents and the field experiments. XX and DC carried out metal ion binding analysis. HS and YP contributed to interpretation and modification of the data and manuscript. All authors read and approved the final manuscript.

## Conflict of interest

The authors declare no conflict of interest.
